# Symptomatic or asymptomatic SAR-CoV-2 positive divers should be medically evaluated before returning to scuba diving

**DOI:** 10.3389/fphys.2022.1022370

**Published:** 2022-11-11

**Authors:** Jean Morin, Nicolas Vallée, Pierre-Louis Dufresne, Sarah Rives, Henri Lehot, Lucille Daubresse, Romain Roffi, Arnaud Druelle, Pierre-Julien Cungi, Jean-Eric Blatteau

**Affiliations:** ^1^ Department of Diving and Hyperbaric Medicine, Sainte-Anne Military Hospital, Toulon, France; ^2^ Military Institute of Biomedical Research (IRBA), Subaquatic Operational Research Team (ERRSO) at Toulon, Bretigny sur Orge, France; ^3^ Department of Intensive Care, Sainte-Anne Military Hospital at Toulon, Toulon, France

**Keywords:** diving, COVID-19, coronavirus, immersion pulmonary edema, pulmonary barotrauma, decompression sicknes, spirometry, chest CT scan

## Abstract

**Introduction:** In order to allow the resumption of diving activities after a COVID-19 infection, French military divers are required to undergo a medical fitness to dive (FTD) assessment. We present here the results of this medical evaluation performed 1 month after the infection.

**Methods:** We retrospectively analyzed between April 2020 and February 2021 200 records of divers suspected of COVID-19 contamination. Data collected included physical examination, ECG, blood biochemistry, chest CT scan and spirometry.

**Results:** 145 PCR-positive subjects were included, representing 8.5% of the total population of French military divers. Two divers were hospitalized, one for pericarditis and the other for non-hypoxemic pneumonia. For the other 143 divers, physical examination, electrocardiogram and blood biology showed no abnormalities. However 5 divers (3.4%) had persistent subjective symptoms including fatigability, exertional dyspnea, dysesthesias and anosmia. 41 subjects (29%) had significant decreases in forced expiratory flows at 25–75% and 50% on spirometry (n = 20) or bilateral ground-glass opacities on chest CT scan (*n* = 24). Only 3 subjects were affected on both spirometry and chest CT. 45% of these abnormalities were found in subjects who were initially asymptomatic or had non-respiratory symptoms. In case of abnormalities, normalization was obtained within 3 months. The median time to return to diving was 45 days (IQR 30, 64).

**Conclusion:** Our study confirms the need for standardized follow-up in all divers after COVID-19 infection and for maintaining a rest period before resuming diving activities.

## Introduction

Since 2020, the highly pathogenic SARS-CoV-2 virus, commonly referred to as COVID-19, has been responsible for a global pandemic with a clinical form of severe acute respiratory syndrome that can affect the entire population (Guan et al., 2020; Zhou et al., 2020). In addition, COVID-19 infection may also manifest as cardiac, digestive, renal or neurological disorders. Thromboembolic complications and myocardial damage are also reported (Chen et al., 2020; Long et al., 2020). Published data most often concern severe forms in patients with comorbidities.

In young, athletic, healthy subjects with asymptomatic or mild COVID-19 infection, the risk of cardiorespiratory complications is not clearly identified because of limited series and contradictory results. The presence of post-COVID clinical manifestations or paraclinical abnormalities does not always correlate with the initial severity of the disease, especially in young subjects who have initial infections related to COVID-19 with few or no symptoms (Daniels et al., 2021; Gervasi et al., 2021; Shaw et al., 2021).

After a COVID-19 infection, the resumption of sports activities generates numerous debates with various recommendations (Phelan et al., 2020; Wilson et al., 2020). The practice of diving, especially in a professional context, raises the same questions concerning the period of resumption and the required level of medical examinations. Indeed, the practice of scuba diving places particular demands on cardiorespiratory function (Tezlaff et al., 2005). The ventilation of dense gas associated with the physical effort constitute well-identified physiological constraints as does immersion, which leads to an increase in venous return (Wilmshurst 2019). Negative pressure ventilation with the different types of diving apparatus and the position of the diver also increase the respiratory work (Castagna et al., 2018). In addition, the cardiorespiratory system is directly involved in the removal of bubbles formed during decompression (Francis and Mitchell 2003). Considering that a pro-inflammatory state or subclinical lesions may persist in a post-COVID setting (Nalbadian et al., 2021), we wonder whether this might promote the occurrence of specific diving accidents such as immersion pulmonary edema, pulmonary barotrauma or decompression sickness.

In order to resume scuba diving activities after COVID-19 infection, it seems essential to verify the absence of residual physical and cardiorespiratory damage, in accordance with the recommendations of learned societies in this field (Diving Medical Advisory Committee, 2022; Divers alert network, 2022; European Underwater and Baromedical Society, 2022; International Association of Francophone Hyperbaric Centres, 2022). However, the first recommendations issued at the beginning of the pandemic were based on limited knowledge with rather heterogeneous proposals.

In this context, the French military health service implemented a mandatory medical evaluation procedure to authorize the resumption of military diving after a documented COVID-19 infection. French military divers are professional divers who benefit from specific medical follow-up including initial and annual fitness to dive (FTD) assessments. This follow-up includes in particular a low-dose chest CT scan at the initial visit, and spirometry at each visit. Following sick leave, divers are usually given an additional visit and appropriate tests to enable them to return to work. This support was also provided to divers who tested positive for SARs-COV-2 PCR. During the period of the study the military divers were not yet vaccinated, the vaccine was mandatory only from the second half of 2021.

This article presents the main results of this specific post-COVID FTD assessment, including the changes observed in the respiratory system.

To the best of our knowledge, there are no publications concerning the evolution of respiratory parameters in the medium term in professional military divers with a mild or non-symptomatic form of COVID-19 infection.

## Methods

This was a single-center retrospective analysis of prospectively collected data of anonymized records over the period from April 2020 to February 2021. The files were all from the hyperbaric medicine and diving expertise department of the Sainte-Anne military hospital at Toulon, the referral center for the expertise of military divers in France. The analysis of these files was also carried out by the same team. They identified the accessible files including all the medical information requested in the framework of the follow-up of divers for the resumption of diving after a COVID-19 infection.

### FTD assessment

The mandatory medical evaluation procedure for resumption of military diving after COVID-19 infection distinguished several situations:

- Diver with confirmed COVID-19 infection with hospitalization;
- Symptomatic diver with a positive PCR test who did not require hospitalization;
- Asymptomatic diver with a positive PCR test.

In the first case, the FTD assessment was performed at the reference center of diving medicine located at the Sainte-Anne military hospital de Toulon, France, 6 months after the end of the hospitalization. In the other cases, the FTD assessment was carried out by the diver’s referring physician who had to send the results to the reference center in Toulon for validation of the resumption of diving. This physician had to describe the symptoms and distinguish between “respiratory” (group 1) and “non respiratory” clinical forms (group 2), as well as asymptomatic subjects (group 3). This FTD assessment was performed at least 1 month after the end of symptoms and at least 14 days for asymptomatic subjects with a positive PCR test.

The medical evaluation included, in addition to the clinical examination, the following tests:

- Measurement of blood pressure, heart rate, room air SpO2 with simple exercise test (step test or walking test);
- Spirometry;
- Electrocardiogram;
- Routine biological check-up;
- Low-dose chest CT scan.

The FTD assessment procedure and its evaluation have been validated by the French Army Health Service.

### Data collection

Data collected for each subject included FTD assessment results with age, biometric data, duration of symptoms, and type of symptoms. Results of additional tests included:

- Blood sample: complete blood count, platelets, C-reactive protein (CRP), creatinine with calculation of glomerular filtration rate;
- Electrocardiogram (ECG);
- Spirometry: forced vital capacity (FVC, L), forced expiratory volume in one second (FEV1, L. sec -1), forced expiratory flow rates (FEF 25–75%,75%,50%,25%, L. sec-1) and ratio FEV1/FVC. All values were expressed as absolute values and as a percentage of the mean values predicted by the European Respiratory Society for standardized pulmonary function tests (Graham et al., 2019). For spirometry, post-COVID results were compared with pre-COVID assessments. The reference values used in the French medical assessment for military diving are those of the CECA-1993 (Quanjer et al., 1993). Values were considered abnormal for measured FEV1/theoretical FEV1 less than 0.9, measured FEF/theoretical FEF less than 0.75, and FEV1/FVC less than 0.75.
- Chest CT scan: Images were obtained using a low-dose GE Revolution CT multilayer scanner. Pathologic findings were analyzed in terms of the presence of ground-glass opacities, fibrous sequelae, reticulations, pneumatoceles, bronchiectasis, mucus plugs, parenchymal lesions, and others (crazy paving pattern; nodules; pleural thickening or pleural effusion).

### Statistical analysis

All the statistical analysis performed with R statistical software. Demographic and descriptive data were expressed as mean (standard deviation, SD), median (interquartile range, IQR) or numbers and percentages where appropriate. Normality of distribution was checked before performing paired or unpaired parametric t tests to analyze spirometry on all 143 subjects or between the 3 groups of divers. For the 20 subjects with abnormal spirometry, a comparison of baseline, 1-month FTD assessment and 3-month control values was performed using the Kruskal–Wallis test followed by the Bonferroni-Dunn post hoc test. The level of significance was set at *p* < 0.05.

## Results

### Population characteristics

Two hundred military divers were tested for COVID-19 infection between March 2020 and February 2021. Fifty-seven records were excluded due to missing data or negative PCR tests. Data from the remaining 145 records are examined in this study, representing approximately 8.5% of the total French military diver population.

All participants were male with a mean age of 33.3 ± 7.4 years and a BMI of 24.8 ± 3.2.21% of subjects had a history of smoking and 9% were active smokers. During the study period, military divers were not yet vaccinated.

145 PCR-positive subjects were included, representing 8.5% of the total population of French military divers. 143 divers were not hospitalized with 52 divers (36%) showing initial respiratory symptoms with dyspnea and cough (group 1), 70 divers (49%) showing only flu-like symptoms (fever, fatigue, body and muscle aches, headache, sore throat) and/or neurological disturbances such as loss of sense of smell/taste (group 2). Finally, 21 divers (15%) were asymptomatic with a positive PCR test (group 3). The duration of symptoms and time to return to diving are specified in Table 1.

**TABLE 1:** General characteristics.

	Total	Group 1	Group 2	Group 3
Initial symptoms	—	Respiratory	Non respiratory	Asymptomatic
Number of subjects, (% of total)	143	52 (36%)	70 (49%)	21 (15%)
Age, in years (mean, SD)	33.3 ± 7.4	35.4 ± 6.9	32.2 ± 6.9	32.5 ± 6.8
Duration of symptoms, in days (mean, SD)	8.5 ± 4.6	10 ± 7.5	7.4 ± 6.7	0
N° of subjects with abnormalities on spirometry (% of subgroup)	20 (14%)	11 (55%)	4 (20%)	5 (25%)
N° of subjects with covid-related images on chest CT scan (% of subgroup)	24 (16.8%)	13 (54%)	6 (25%)	5 (21%)
Time to return to diving, in days (median, IQR)	45 (30, 64)	50 (42, 71)	46 (30, 71)	30 (21, 31)

Two divers were hospitalized, one for pericarditis and the other for non-hypoxemic pneumonia (Table 2).

**TABLE 2:** Description of the 7 cases of divers with persistent subjective symptoms.

Age, years	Initial COVID symptoms	Post-COVID symptoms	Chest CT scan abnormalities	Spirometry abnormalities	Return to dive, days
22	Pericarditis, 1 week **hospital stay**	Fatigability for 3 months	no	no	254
41	Non-hypoxemic pneumonia, 1 week **hospital stay**	Fatigability for 1 month	Ground glass 10%	Decreased in FEF 25–75%	75
32	Respiratory symptoms for 2 weeks	Upper limb dysesthesias, fatigability and headache for 1 month	Ground glass 10%	no	114
35	Non respiratory symptoms, 1 week	Anosmia for 2 months	no	no	90
38	Respiratory symptoms, 1 week	Feeling of discomfort during physical effort for 2 months, normal exercise test	no	no	90
38	Non respiratory symptoms, 1 week	Fatigue for 2 months	no	no	129
40	Respiratory symptoms, 1 week	Shortness of breath on exertion, headache for 2 weeks	Limited ground glass	no	30

### Post-COVID examinations

Patient interview and clinical examination revealed that in addition to the two hospitalized subjects, 5/143 other divers (3.4%) had persistent symptoms including prolonged fatigue, exertional dyspnoea, dysesthesias and anosmia (Table 2).

No abnormalities were observed on electrocardiogram, urinalysis and blood biology compared to previous FTD assessments.

Only one subject showed clinical symptoms that could meet the World Health Organization definition of a long COVID syndrome including shortness of breath on exertion or fatigability or neuropsychological changes persisting for more than 2 months. This was a case of pericarditis which had resulted in hospitalization with a period of significant fatigability for 3 months (Table 2).

### Spirometry

All baseline spirometry (pre-COVID) was within medical standards for military divers. There was no difference between groups on baseline spirometry values. There was no significant difference between spirometry before and after COVID-19 infection for the overall population (*n* = 143) or in the subgroup analysis (*n* = 52/70/21).

**FIGURE 1:**
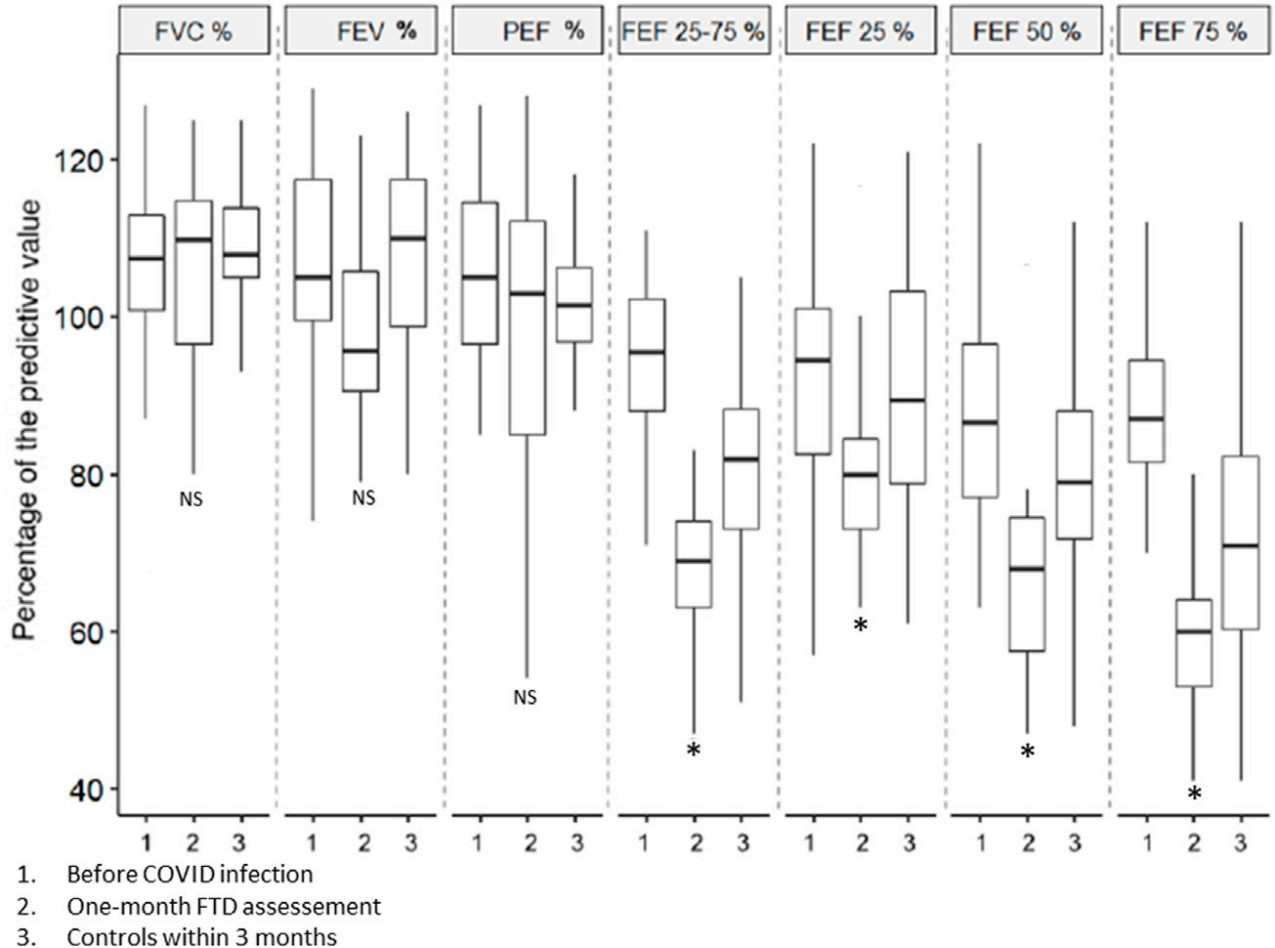
Evolution of spirometry values for the 20 subjects who had abnormalities on the post-COVID FTD assessment. The figure shows the baseline mean values (1), the values during the FTD assessment at 1 month (2) and the control performed within 3 months (3). Error bars represent standard error of the mean. * represents a significant difference from baseline values.

However, from a clinical point of view, 20 subjects (14%) had abnormal spirometric values on post-COVID examination. Of these, 11 (55%) were in group 1; 4 (20%) in group 2 and 5 (25%) in group 3 (Table 1). Abnormalities predominated significantly in the distal part of the respiratory system i.e. 50% and 75% FEF with a mean FEF 50% reduced to 66.2 ± 17.7% of the theoretical value and a mean FEF 25–75% to 65.6 ± 13.7% of the predicted value. Due to these abnormalities, these subjects had a follow-up spirometry within 3 months, with secondary normalisation of the initially decreased FEF values. (Figure 1).

### Chest CT scan

**FIGURE 2:**
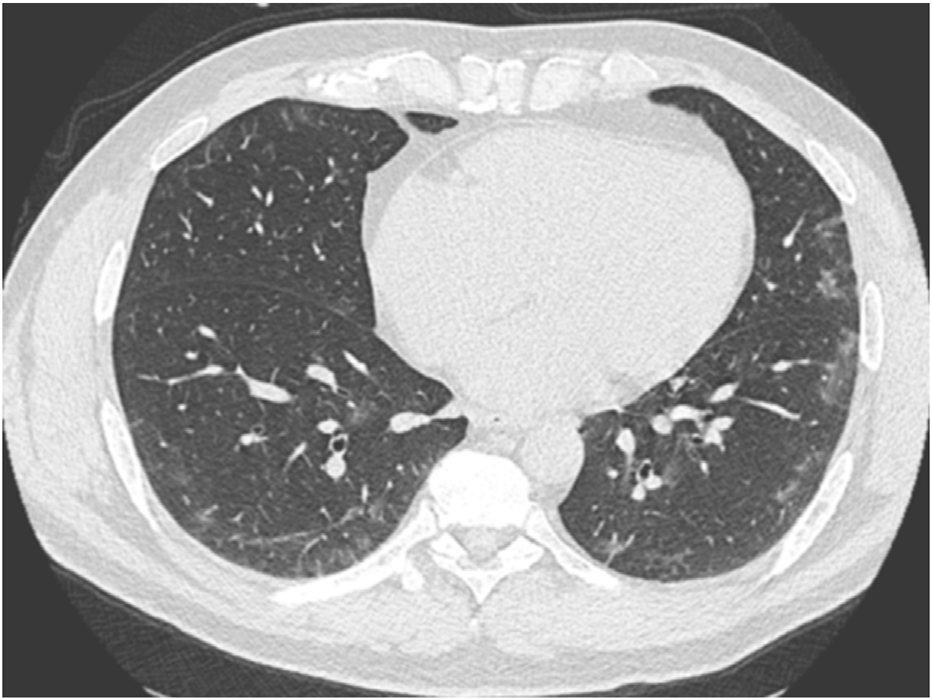
Asymptomatic diver with a chest CT scan performed 1 month after a positive PCR test. A characteristic appearance of very mild COVID-19-related lung involvement with limited bilateral ground glass opacities can be noted.

Twenty-four subjects (16.8%) had COVID-induced pulmonary injury on follow-up chest CT. 13 (54%) were in group 1, 6 (25%) in group 2, and 5 (21%) in group 3 (Table 1). The lesions affected less than 10% of the lung volume and were bilateral. They were non-systematized ground-glass opacities (Figure 2), with a sub pleural localization, mainly in the basal areas. Of these subjects, only 3 also had abnormal spirometry. To ensure that the lesions had disappeared, a follow-up CT scan was systematically performed within 3 months after the first CT scan.

CT scans showed non-covid-related “incidentaloma” abnormalities in 21 subjects (14.7%). With the exception of a single case of diagnosed sarcoidosis, the majority of lung abnormalities were benign nodules in 13 subjects, mostly requiring no further monitoring. Other abnormalities were found notably at the subdiaphragmatic level with renal, hepatic, pancreatic and adrenal cysts all benign. There was also the discovery of a coronary calcification in one subject and an upper posterolateral tracheal diverticulum in another diver.

These abnormalities had not been noted until now because these subjects had not had a CT chest scan at their initial FTD examination, as this is only mandatory since 2018.

### Unfitness to dive

The median (IQR) time to return to diving was 45 (30, 64) days, with differences between the groups from 30 days for the group 3–50 days for group 1 (Table 1).

The median time for the 20 subjects with abnormal spirometry was 57 (33, 74) days while the median time for the 24 subjects with abnormal CT was 67 (60, 90) days.

Dive resumption times for symptomatic subjects are specified in Table 2.

Only one case of permanent unfitness to dive was pronounced after the incidental discovery of pulmonary sarcoidosis.

## Discussion

The main result of this study is the demonstration of persistent abnormalities on spirometry or lung CT in subjects with symptomatic respiratory forms, others symptomatic but without respiratory signs and also asymptomatic subjects. These data confirm the interest of pursuing a FTD assessment with complementary respiratory examinations for the whole population of divers and not only those who are symptomatic with respiratory signs.

Indeed, the absence of clinical signs of severity and a good clinical recovery do not necessarily imply the absence of spirometric or CT chest scan abnormalities found in 14 and 17% of cases, respectively. It is worth mentioning that the majority of subjects with CT abnormalities are not the same as those with spirometric abnormalities. This justifies the performance of these two examinations which are complementary to explore respiratory problems.

The abnormalities found on spirometry correspond to a small airway disease which is a subclinical syndrome defined by a decrease in FEF 25–75%, FEF 25% and FEF 50% (Allen et al., 2010). The origin is multifactorial and may be due to a history of childhood asthma, a pulmonary infectious episode or exposure to toxicants (tobacco, pollutants). It can regress, stabilize or evolve into an obstructive respiratory disorder. It is important to remember that all divers in this study had baseline values that were within the normal range on spirometry and that the decrease in FEFs was only seen in the context of the FTD assesment post-COVID. The medical consequences of these spirometric anomalies during diving are not known. The respiratory resistances which increase in parallel with the increase in gas density and therefore in depth lead to a significant decrease in FEV1 and also accentuate the decrease in FEFs, leading to pseudo-obstructive breathing. The reduction of respiratory flow in the small airways and the respiratory effort during diving could thus favor the occurrence of immersion pulmonary edema or pulmonary barotrauma (Tezlaff et al., 1997; Wilmshurst 2019).

In addition, chest CT scans have shown abnormalities in patients who were asymptomatic at the time of the FTD assessment. These ground-glass abnormalities were suggestive of lesions associated with COVID-19 infection, but were also quite similar to lesions seen in immersion pulmonary edema. Therefore, it is legitimate to question the possible consequences of exposure to immersion in this context of subclinical lung injury. These CT abnormalities are probably a risk factor for pulmonary barotraumas during diving. However, indeed, we cannot calculate the risk.

Approximately 8.5% of the total population of French military divers were tested positive for COVID-19 during the study period.

With the exception of the two hospitalized subjects, most of the other 143 (49%) had a non-respiratory form, 36% had a respiratory form and 15% were asymptomatic. The study population consisted of mild to moderate forms in military athletes aged 33 ± 7 years with no comorbidities.

Some subjects who had a normal physical examination described subjective signs persisting at the time of the FTD assessment. The majority of symptoms were consistent with those described in a post-covid setting including fatigability, exertional dyspnea, dysesthesias and anosmia (Nalbandian et al., 2021). The only case of long covid that met the WHO definition with signs persisting for more than 2 months was the case of pericarditis, which presented with fatigability lasting for 3 months.

In our study, the resumption of diving was rather late, about 55 days on average after the beginning of the infection, whatever the clinical form. It is obvious that subjects who were fully asymptomatic with normal investigations were able to return to diving more quickly than those who had paraclinical abnormalities that needed to be monitored. The average time to return to diving is therefore directly influenced by the 29% of subjects who required a check-up after the discovery of an abnormality on spirometry or CT scan. Normalization has been observed within 3 months after controlling spirometry or CT scan. Therefore, this 3-month period to verify normalisation and authorise resumption of diving seems reasonable and consistent with the literature, including the data from the Turkish study which even recommends a period of at least 3 months for resumption of diving in the case of abnormalities initially discovered on chest CT scan (Mirasoglu et al., 2022).

Our results are partially consistent with the initial recommendations of the learned societies. The European Underwater and Baromedical Society and the European Committee of Hyperbaric Medicine have indeed issued recommendations for recreational and professional diving after the COVID-19 outbreak (International Association of Francophone Hyperbaric Centres, 2022). They recommended that divers who remained asymptomatic should wait at least 1 month before resuming diving and that divers who developed symptoms of COVID-19 but did not require hospitalization should wait at least 3 months before seeking a diving clearance from a diving medicine specialist. In our series we have in fact allowed the return to diving faster than 3 months for the subjects presenting no abnormality during the FTD assessment.

Since the beginning of 2022, the evolution of the virus with the emergence of new variants such as the omicron variant as well as mass vaccination campaigns have changed the clinical presentation with a large number of minor forms with rapidly resolving general or ENT signs in young subjects. Thus, the initial recommendations have been modified in France to allow the resumption of military diving activities more rapidly in case of a minor (14 days) or asymptomatic form (7 days). However, the delay of 1 month before the FTD assessment has been maintained for respiratory forms with prescription of a chest CT scan and spirometry. In case of abnormality, a control is necessary at 3 months with a resumption of the diving after normalization. To date, we have not observed an increase in the number of diving accidents, especially in subjects previously infected with COVID-19.

This study has some limitations. First, because it is a retrospective study, the level of evidence that can be expected is lower than that of a prospective study. Second, the study population is highly selective, consisting of young, athletic, healthy military subjects. Indeed, military diver candidates are not representative of the general population of professional or recreational civilian divers. It is worth mentioning that spirometry and CT scans do not evaluate all respiratory problems. However, we have chosen to focus only on these two tests because they are non-invasive and are part of the “standard” workup for the FTD assessment in military divers. Furthermore, even if we did not observe any abnormality on the ECG or during simple stress tests with pulse oximetry measurement, this does not exclude the possibility of cardio-respiratory abnormalities such as reported in the literature for athletes, based on cardiac biomarkers or further adjunctive testing with cardiac magnetic resonance imaging, exercise testing, or ambulatory rhythm monitoring (Phelan et al., 2019). The post-covid evaluation of the cardio-respiratory system, particularly during exercise, is a strong recommendation for the majority of learned societies.

## Conclusion

A post COVID FTD assessment was set up at the beginning of the pandemic for French military divers who were not vaccinated at that time. The results are reassuring with 8.5% of positive cases, and relatively few cases with persistent subjective signs. The main finding was the discovery of 29% of subjects who were not symptomatic at the time of the FTD assessment, but who had respiratory abnormalities on spirometry or CT scan. Abnormal spirometry showed decreases in 25–75% forced expiratory flows, while chest CT scans showed bilateral ground glass opacities. Our study suggests that if an abnormality is detected, normalization is observed within 3 months. We suggest that it is prudent to wait for the normalization of paraclinical examinations before resuming diving. Indeed, the consequences of the presence of these anomalies are not known with a potential risk of bronchial hyper reactivity or subclinical alveolar lesions which could favor the occurrence of diving accidents.

No military diver has had a diving accident after infection with COVID-19 within the prescribed period.

It seems essential to us to continue to carry out a post-covid FTD assessment which should not however be limited to spirometry or a chest CT scan, but by carrying out a complete medical examination with the evaluation of tolerance during of physical exertion. Given the continued evolution of COVID-19 and the strategies for its control, it seems appropriate to continue recording medical data to guide future recommendations by learned societies in this field.

## Data Availability

The raw data supporting the conclusions of this article will be made available by the authors, without undue reservation.
